# Clinical characteristics, management, and outcomes of lacrimal gland adenoid cystic carcinoma: a single-center series with contextual comparison to SEER data

**DOI:** 10.3389/fonc.2026.1900973

**Published:** 2026-09-17

**Authors:** Yongbo Xiang, Ping Zhou, Yingming Zhu, Xiaoshuai Bai, Junlin Yi, Jingwei Luo, Xiaodong Huang, Kai Wang, Yuan Qu, Xuesong Chen, Runye Wu, Jinbo Wang, Ye Zhang

**Affiliations:** 1 Department of Radiation Oncology, National Cancer Center/National Clinical Research Center for Cancer/Cancer Hospital, Chinese Academy of Medical Sciences and Peking Union Medical College, Beijing, China; 2 Department of Oncology, Qingdao Central Hospital, University of Health and Rehabilitation Sciences (Qingdao Central Hospital), Qingdao, China; 3 Department of Emergency Internal Medicine, the Affiliated Hospital of Qingdao University, Qingdao, China; 4 Qingdao Medical College, Qingdao University, Qingdao, China

**Keywords:** case series, lacrimal gland adenoid cystic carcinoma, multimodal therapy, radiotherapy, survival, treatment-related toxicity

## Abstract

**Introduction:**

Lacrimal gland adenoid cystic carcinoma (LGACC) is a rare, aggressive malignancy with limited epidemiological data, particularly from East Asian populations.

**Methods:**

This retrospective single-center series describes the clinicopathological features, treatment patterns, and outcomes of six consecutive patients with pathologically confirmed LGACC treated at the Cancer Hospital, Chinese Academy of Medical Sciences between 2012 and 2024. Findings were placed in the context of 22 cases extracted from the SEER database (ICD-O-3 histology code 8200/3, primary site C69.6). Survival was estimated for the institutional cohort and, descriptively, for a combined regional-stage cohort.

**Results:**

The median age at diagnosis was 31 years (range 26–59), with equal sex distribution. All tumors were regional-stage, and 66.7% were T3–4. All patients underwent surgery (orbital exenteration in 66.7%, globe-sparing resection in 33.3%; R0 margins in 33.3%) followed by high-dose conformal radiotherapy (66–70 Gy). One disease-related death occurred. Five-year overall survival (OS) and disease-specific survival (DSS) were both 75% (95% CI 42.6–100%). Local recurrence was observed in 16.7% and distant metastases in 50%. Treatment-related toxicity was mostly grade I–II. In a combined regional-stage cohort of 17 patients, surgery plus radiotherapy was associated with numerically higher 5-year OS (64.8% versus 26.7%) and DSS (74.1% versus 26.7%) compared with surgery or radiotherapy alone; these estimates are presented only for descriptive context and are limited by small numbers and wide confidence intervals. A sensitivity analysis excluding two SEER patients with unknown surgery status who received radiotherapy alone yielded consistent numerical trends. Chemotherapy was not used as primary or adjuvant therapy for the initial disease; it was given concurrently with radiotherapy for early postoperative residual disease in one patient and after delayed local recurrence in another.

**Discussion:**

In this series of predominantly advanced LGACC, surgery followed by adjuvant high-dose conformal radiotherapy achieved favorable local control with predominantly mild toxicity. Combined SEER comparisons should be interpreted cautiously because of the small sample size.

## Introduction

Lacrimal gland adenoid cystic carcinoma (LGACC) is a rare and aggressive malignancy originating from the secretory epithelial cells of the lacrimal gland and represents the most common malignant epithelial tumor at this site (1). In the United States, approximately 25 new cases are diagnosed annually, accounting for about 1.5% of all adenoid cystic carcinomas (ACCs) (2). Although the global incidence of ACC ranges from 3 to 4.5 cases per million population and occurs predominantly in salivary glands, the lacrimal gland variant carries a substantially worse prognosis, with mortality rates approximately 3.5 times higher than those of other ACC subtypes (3). Despite multimodal therapy, 5-year overall survival (OS) rates remain only 60–70% and decline further to 40–50% at 10 years, largely driven by high rates of perineural invasion (up to 70%) and distant metastasis (30–50%) (4).

Current epidemiological and clinical insights into LGACC are limited and derived predominantly from Western populations, particularly the Surveillance, Epidemiology, and End Results (SEER) database, which covers approximately 28% of the U.S. population (5, 6). Survival outcomes vary according to tumor size and local invasiveness; however, SEER lacks molecular data and is subject to Western demographic bias, thereby limiting its global applicability (7). Although ACC is rare (approximate incidence 3–4.5 cases per million) and robust worldwide comparative data remain limited, population-based studies from the United States have demonstrated a declining trend in the incidence of head and neck ACC over recent decades (8, 9). Data from Asian populations—especially China—remain sparse and are largely restricted to single-center reports (10). Recent Chinese multicenter observations suggest a younger age at onset and greater local aggressiveness in Asian cohorts, raising the possibility of regional differences influenced by genetic and environmental factors (11).

Although recent systematic reviews and meta-analyses, including the comprehensive analysis by Yan et al. that pooled 30 studies involving 585 patients (12), have synthesized global clinicopathological features and confirmed the superiority of multimodal therapy over surgery alone, high-quality real-world evidence from East Asian populations is still scarce. In particular, detailed data on modern intensity-modulated radiotherapy techniques [volumetric modulated arc therapy (VMAT) and helical tomotherapy (TOMO)], rigorous ocular dose constraints guided by Quantitative Analysis of Normal Tissue Effects in the Clinic (QUANTEC) recommendations, structured long-term visual outcomes, and direct comparison with contemporary SEER data in patients treated at high-volume Chinese centers are still lacking.

This single-center series of six consecutive patients with pathologically confirmed LGACC treated at the Cancer Hospital of the Chinese Academy of Medical Sciences (CHCAMS) between 2012 and 2024 addresses these gaps. We provide complete demographic, clinical, imaging, and pathologic characteristics; detailed radiotherapy planning parameters with dedicated ocular-protection protocols; treatment-related toxicity profiles; and survival outcomes, and place these institutional findings in the context of SEER data. The principal contribution of the present work lies in the granular reporting of contemporary conformal radiotherapy delivery, QUANTEC-guided organ-at-risk sparing, and long-term visual function in an East Asian cohort composed predominantly of advanced-stage disease—elements that remain sparsely documented in the existing literature—and in the confirmation of a younger age at onset and marked local aggressiveness relative to Western population-based data. Together, these observations offer practical real-world evidence to support optimized multimodal management strategies for LGACC in the Asian setting.

## Materials and methods

### Study design and patients

This retrospective single-center case series included six consecutive patients with pathologically confirmed lacrimal gland adenoid cystic carcinoma (LGACC) who were treated at the Cancer Hospital of the Chinese Academy of Medical Sciences (CHCAMS) in Beijing between April 2012 and April 2024. All diagnoses were confirmed by histopathological examination of surgical specimens. Clinical data were extracted from electronic medical records, supplemented by telephone follow-up and death registry data. Collected variables included demographics, clinical presentation, tumor characteristics, treatment details, treatment-related complications, recurrence, metastasis, and survival outcomes. Overall survival (OS) was defined as the time from diagnosis to death from any cause, and disease-specific survival (DSS) was defined as the time from diagnosis to death attributable to LGACC. Survival times were calculated in months from the date of diagnosis to death or last follow-up. The final follow-up date was May 31, 2025, with a median follow-up of 60 months.

### SEER data

Data from the U.S. Surveillance, Epidemiology, and End Results (SEER) 17 registries (2001–2022) were used solely as a contextual reference and historical benchmark for demographic patterns and survival estimates in LGACC. Cases were identified using SEER*Stat software (version 8.4.3) with the International Classification of Diseases for Oncology, Third Edition (ICD-O-3) histology code 8200/3 and primary site code C69.6, yielding 22 patients. Baseline characteristics, tumor attributes, treatment modalities, and survival information (including cause of death) were extracted. T category was retrospectively assigned based on extent-of-disease and collaborative staging codes according to the American Joint Committee on Cancer (AJCC) 8th edition criteria when feasible. Patients were stratified by tumor stage using the SEER Historic Stage A schema. Survival status and cause of death were derived from linked vital records, with a median follow-up of 50.5 months.

It should be noted that the SEER database provides limited clinical and treatment granularity. Detailed information on presenting symptoms, imaging findings, precise pathologic features (including Ki-67 index and MYB rearrangement status), radiation technique, total dose, fractionation schedule, ocular dose constraints, treatment-related toxicity, and long-term visual outcomes is not available. Radiation and chemotherapy are recorded only as “yes” versus “no/unknown,” with known incomplete ascertainment.

### Statistical analysis

Normally distributed continuous variables were summarized as means with standard deviations, and non-normally distributed variables as medians with interquartile ranges (IQR). Categorical variables were presented as frequencies and percentages. OS and DSS were estimated using the Kaplan–Meier method for descriptive purposes only. Given the small institutional sample size and inherent limitations of SEER data, no formal comparative statistical testing (including log-rank tests) was performed. SEER data served exclusively as a contextual reference. All analyses were conducted using SPSS version 27.0 (IBM) and R software (version 4.2.2).

### Ethics statement

The single-center series was approved by the Ethics Committee of the Cancer Hospital of the Chinese Academy of Medical Sciences (approval number: 25/736-5683). The requirement for informed consent for the retrospective review of medical records was waived due to the retrospective nature of the study. All patient data presented in this manuscript have been de-identified. Individual-level details in **Table 1** are provided only after removal of potentially identifying combinations of rare clinical features. Written informed consent for publication of de-identified clinical details was not required by the ethics committee for this retrospective series. Analysis of publicly available SEER data was exempt from institutional review board approval.

**Table 1:** Detailed clinical, pathological, treatment, and outcome characteristics of the six patients with lacrimal gland adenoid cystic carcinoma (LGACC) treated at CHCAMS.

Patient	Age/sex	Side	Symptoms	Size (cm)	Invasion	T stage	Surgery	Margin	Radiotherapy	Key pathology	Metastasis (site/time)	Survival (mo)/status
1	35/F	Right	Proptosis, diplopia, ↓VA	4.0	EOM + ON	T3	Orbital exenteration	Non-R0	TOMO 69.96 Gy/33 fx	Ki-67 20%	None	116/Alive
2	30/F	Left	Proptosis, pain	2.2	None	T2	Complete excision	R0	VMAT 66 Gy/30 fx	—	None	92/Alive
3	28/M	Right	Upper eyelid ptosis	2.7	EOM	T2	Complete excision	R0	VMAT 69.96 Gy/33 fx (after recurrence)	CK+, CK5/6+, CD117+	Lung/91 mo	157/Alive
4	59/M	Right	Proptosis, pain	4.3	Bone + ON	T4	Orbital exenteration	Non-R0	VMAT 69.96 Gy/33 fx	Ki-67 25%, CK7+, CD117+	Bone/10 mo	28/Dead
5	31/M	Right	Proptosis	4.3	Bone + ON	T4	Orbital exenteration	Non-R0	VMAT 69.96 Gy/33 fx	Ki-67 ~15%, MYB rearrangement	None	26/Alive
6	26/F	Right	Proptosis, diplopia, pain	4.8	EOM + Bone + ON	T4	Orbital exenteration	Non-R0	VMAT 66 Gy/30 fx + concurrent lobaplatin	Ki-67 40%, CK7 (3+), CD117 (2+)	Bone/10 mo	13/Alive

All patients received radiotherapy with strict ocular dose constraints according to QUANTEC guidelines. No severe ocular toxicity was observed. Long-term visual function was preserved in five of six patients (83.3%).

EOM, extraocular muscle; ON, optic nerve; VA, visual acuity; VMAT, volumetric modulated arc therapy; TOMO, helical tomotherapy; fx, fractions.

## Results

### Clinical characteristics of patients in the CHCAMS case series

Six consecutive patients with pathologically confirmed LGACC were included (**Table 1**). ECOG performance status was 0 in one patient (16.7%), 1 in four patients (66.7%), and 2 in one patient (16.7%). Age at diagnosis ranged from 26 to 59 years, with a median of 31 years (IQR 28–35); five patients (83.3%) were younger than 40 years. The series comprised three females and three males. All patients were of Asian ethnicity. The primary tumor was located on the right side in five cases (83.3%) and on the left side in one case (16.7%).

The most common presenting symptoms were proptosis (five patients, 83.3%), pain (three patients, 50.0%), and diplopia (two patients, 33.3%). Reduced visual acuity and upper eyelid ptosis were each observed in one patient (16.7%). Maximum tumor diameter ranged from 2.2 to 4.8 cm (mean 3.7 ± 1.0 cm). Orbital wall bone involvement and extraocular muscle invasion were each present in three patients (50.0%), while optic nerve involvement was identified in four patients (66.7%). According to SEER staging, all six tumors were classified as regional. T category was T2 in two patients (33.3%), T3 in one patient (16.7%), and T4 in three patients (50.0%); all patients were N0. Immunohistochemical findings included a Ki-67 proliferation index ranging from 15% to 40% (mean 25%). One patient harbored a MYB gene rearrangement. Detailed clinicopathological features are presented in **Table 1**.

### Treatment course in the CHCAMS case series

All six patients underwent surgical resection. Complete excision of the lacrimal gland tumor was performed in two patients (33.3%), whereas orbital exenteration was required in four patients (66.7%) because of extensive local disease. Surgical margins were R0 in two patients (33.3%) and non-R0 in four patients (66.7%).

All patients received radiotherapy. Five patients underwent postoperative adjuvant radiotherapy (**Figures 1A, B**), and one patient received radiotherapy after re-excision for local recurrence. Radiotherapy was delivered with volumetric modulated arc therapy (VMAT) in five patients and helical tomotherapy (TOMO) in one patient, to a total dose of 66–70 Gy in 30–33 fractions (**Figures 1C, D**). Strict ocular dose constraints consistent with QUANTEC guidelines were applied, and eyelid retractors were used to minimize ocular toxicity (13, 14). Treatment-related toxicities were generally mild and included grade I–II radiation dermatitis (five patients), grade II–III nasal/oral mucositis (one patient), grade II radiation conjunctivitis (one patient), and maxillary sinus effusion (one patient). No severe ocular toxicities occurred, and long-term visual function was preserved in five of six patients (83.3%) (**Figure 2**).

**Figure 1:**
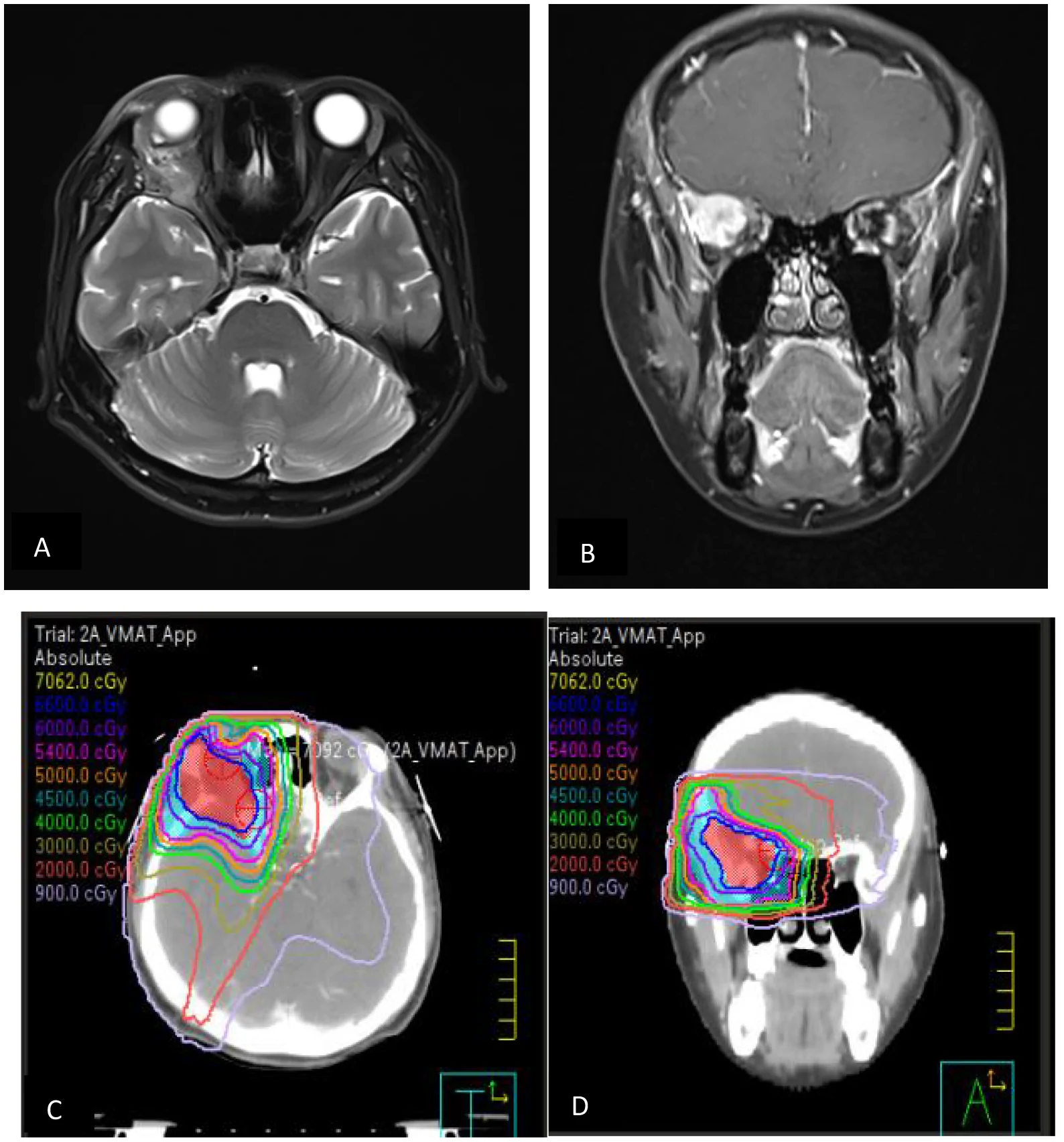
MRI findings of residual tumor following right lacrimal gland ACC surgery and the associated radiation therapy plan. A soft-tissue mass is observed in the postoperative region of the right lacrimal gland, demonstrating mildly hyperintense signal on T2-weighted fat-suppressed images (T2WI/FS) **(A)** and heterogeneous enhancement on contrast-enhanced images **(B)**. The lesion extends to involve the lateral rectus muscle, the superior extraocular muscle group, and the superior and lateral orbital walls. Radiotherapy planning is shown in panels **(C, D)** treatment was delivered using 6-MV X-rays with VMAT. The planned gross tumor volume of the tumor bed (PGTVtb; blue contour line) includes the residual tumor in the postoperative region, with a prescribed total dose of 66 Gy, delivered in 30 fractions.

**Figure 2:**
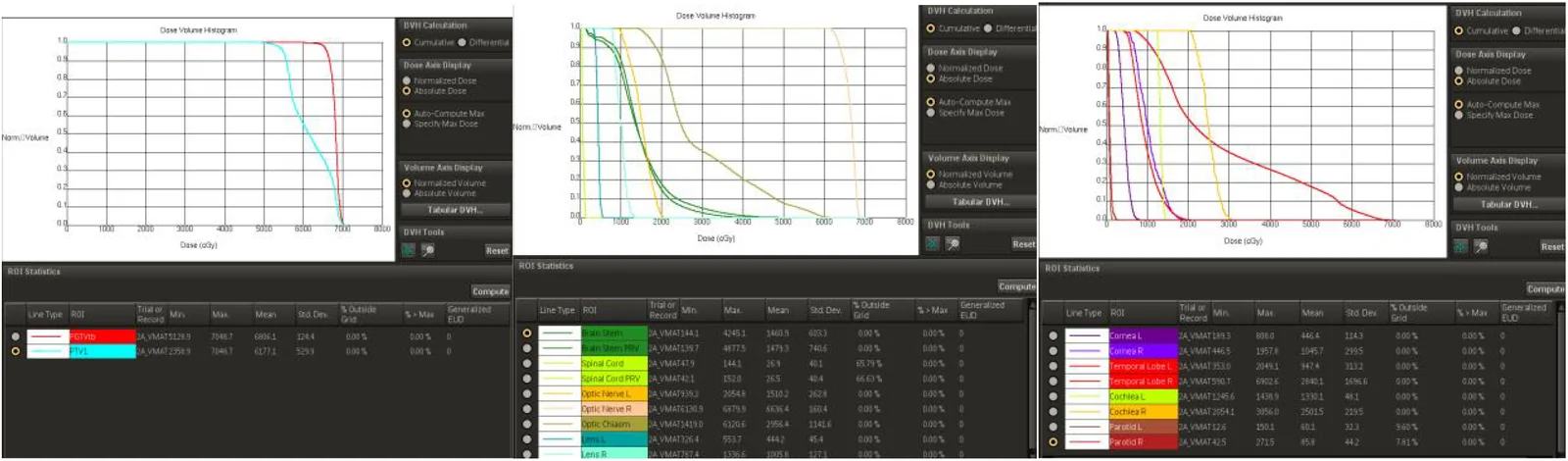
Dose-volume histogram (DVH) of a representative patient with lacrimal gland adenoid cystic carcinoma, illustrating target coverage and ocular sparing. The DVH demonstrates that the PGTVtb (planning gross tumor volume of the tumor bed) and PTV1(planning target volume 1) target volumes received high radiation doses, achieving excellent tumor control with target coverage exceeding 95%. Radiation exposure to ocular structures, including the lens and cornea, was substantially lower (e.g., mean lens dose <10 Gy, corneal dose <40 Gy), well below recommended tolerance thresholds. Although the doses to the optic chiasm and right optic nerve approached their respective tolerance limits, preoperative tumor encasement of the optic nerve accounted for the slightly elevated doses, which nonetheless remained within acceptable safety margins. These results confirm the plan’s effectiveness in providing adequate ocular protection.

No patient received chemotherapy as part of primary treatment. One patient received a single cycle of oxaliplatin plus tegafur after re-excision for delayed local recurrence and before salvage radiotherapy. Another patient received concurrent lobaplatin during radiotherapy for early residual disease.

Local recurrence occurred in one patient (16.7%), who developed an intraorbital relapse approximately 80 months after initial surgery and was managed with re-excision, one cycle of chemotherapy, and subsequent radiotherapy. Distant metastases developed in three patients (50.0%) at 10, 10, and 91 months after primary surgery, involving bone (two patients) and lung (one patient). Post-metastasis management included palliative radiotherapy to bone lesions, bisphosphonates, and anti-angiogenic agents (anlotinib or apatinib). Detailed treatment and outcome information is summarized in **Table 1**.

### Survival and prognosis in the CHCAMS case series

Complete follow-up was available for all six patients (final follow-up date 31 May 2025; median follow-up 60 months). At the time of last follow-up, five patients (83.3%) were alive and one patient (16.7%) had died of disease progression 18 months after the onset of distant metastasis. Median survival was 60 months (IQR 26–116 months; range 13–157 months). Both the 5-year and 10-year overall survival (OS) rates were 75.0% (95% CI 42.6%–100.0%). The corresponding disease-specific survival (DSS) rates were also 75.0% (95% CI 42.6%–100.0%).

### Characteristics of the SEER cohort

The SEER cohort comprised 22 patients (**Table 2**). Median age at diagnosis was 50 years (IQR 37–58 years), and 59.1% of patients were 40 years or older. Males accounted for 63.6% of the cohort. Racial distribution was Black (36.4%), White (36.4%), Asian (22.7%), and Pacific Islander (4.5%). Tumors were left-sided in 59.1% of cases. SEER stage was localized in 40.9%, regional in 50.0%, and distant in 4.5%. T1–2 tumors accounted for 54.5% and T3–4 tumors for 31.8% of cases. Nodal status was N0 in 68.2% and N1 in 4.5%. Surgery was performed in 90.9% of patients, with R0 resection achieved in 45.5%. Radiotherapy was administered in 54.5% and chemotherapy in 9.1% of cases.

**Table 2:** Baseline characteristics of the CHCAMS case series and the SEER cohort.

Characteristic	CHCAMS (n = 6)	SEER (n = 22)
Age, median (IQR), years	31 (28–35)	50 (37–58)
Age < 40 years, n (%)	5 (83.3%)	9 (40.9%)
Age ≥ 40 years, n (%)	1 (16.7%)	13 (59.1%)
Sex, n (%)		
Male	3 (50.0%)	14 (63.6%)
Female	3 (50.0%)	8 (36.4%)
Race, n (%)		
Black	0 (0.0%)	8 (36.4%)
White	0 (0.0%)	8 (36.4%)
Asian	6 (100.0%)	5 (22.7%)
Pacific Islander	0 (0.0%)	1 (4.5%)
Laterality, n (%)		
Left	1 (16.7%)	13 (59.1%)
Right	5 (83.3%)	9 (40.9%)
SEER stage, n (%)		
Localized	0 (0.0%)	9 (40.9%)
Regional	6 (100.0%)	11 (50.0%)
Distant	0 (0.0%)	1 (4.5%)
Unknown	0 (0.0%)	1 (4.5%)
T-stage, n (%)		
T1–2	2 (33.3%)	12 (54.5%)
T3–4	4 (66.7%)	7 (31.8%)
TX	0 (0.0%)	3 (13.6%)
N-stage, n (%)		
N0	6 (100.0%)	15 (68.2%)
N1	0 (0.0%)	1 (4.5%)
Unknown	0 (0.0%)	6 (27.3%)
Surgery performed, n (%)		
Yes	6 (100.0%)	20 (90.9%)
Unknown	0 (0.0%)	2 (9.1%)
Surgical margins, n (%)		
R0	2 (33.3%)	10 (45.5%)
Non-R0	4 (66.7%)	10 (45.5%)
Unknown	0 (0.0%)	2 (9.1%)
Radiation therapy, n (%)		
Yes	6 (100.0%)	12 (54.5%)
No/Unknown	0 (0.0%)	10 (45.5%)
Chemotherapy, n (%)		
Yes	2 (33.3%)	2 (9.1%)
No/Unknown	4 (66.7%)	20 (90.9%)
Survival, median (IQR), months	60 (26–116)	51 (19–117)

Chemotherapy in the CHCAMS series (n=2) was given only in the post-recurrence or early residual setting and was not part of primary therapy.

Median survival in the SEER cohort was 51 months (IQR 19–117 months). The 5-year and 10-year OS rates were 60.6% (95% CI 41.4%–88.6%) and 54.5% (95% CI 35.4%–84.0%), respectively. The corresponding DSS rates were 70.0% (95% CI 50.7%–96.6%) and 63.0% (95% CI 43.0%–92.4%).

### Descriptive analysis of the combined regional-stage cohort

Descriptive comparison of the CHCAMS series (n = 6) and the SEER cohort (n = 22) showed numerically higher survival estimates in the institutional series (5-year OS 75.0% versus 60.6%; 5-year DSS 75.0% versus 70.0%). When the two datasets were combined (n = 28) and restricted to patients with regional-stage disease (n = 17; 60.7% of the combined cohort), Kaplan–Meier curves (**Figures 3A, B**) indicated that surgery combined with radiotherapy was associated with numerically higher 5-year OS [64.8% (95% CI 39.3%–100.0%) versus 26.7% (95% CI 5.07%–100.0%)] and DSS [74.1% (95% CI 48.4%–100.0%) versus 26.7% (95% CI 5.07%–100.0%)] compared with surgery or radiotherapy alone (**Table 3**). Two SEER patients with unknown surgery status who received radiotherapy alone were retained in the non-combined-modality group; a sensitivity analysis excluding these cases yielded consistent numerical trends with wider confidence intervals. These differences are presented for contextual illustration only. Chemotherapy was not used in the primary treatment setting in the institutional series.

**Figure 3:**
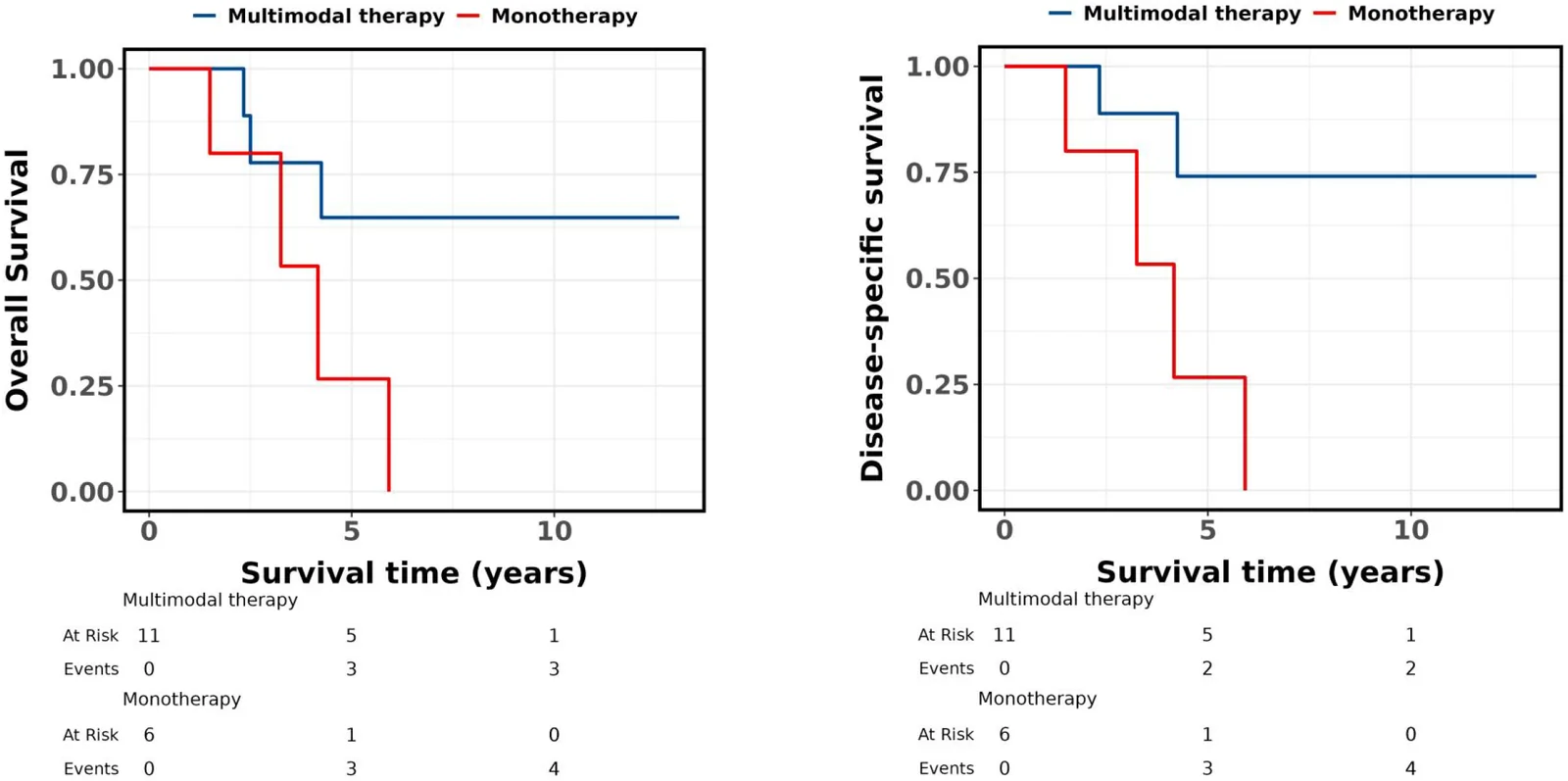
Kaplan–Meier survival curves of regional-stage LGACC patients stratified by treatment modality. Overall survival and disease-specific survival comparing multimodal therapy (surgery + radiotherapy ± chemotherapy) versus monotherapy (surgery or radiotherapy alone). The curves are presented for descriptive purposes only. No formal statistical comparisons were performed. Numbers at risk are shown below each panel.

**Table 3:** Descriptive 5-year overall survival (OS) and disease-specific survival (DSS) estimates by treatment modality in the regional-stage subset.

Treatment modality	N	5-year OS (95% CI)	5-year DSS (95% CI)
Multimodal therapy (Surgery + Radiotherapy ± Chemotherapy)	11	64.8% (39.3–100.0%)	74.1% (48.4–100.0%)
Monotherapy (surgery or radiotherapy alone)	6	26.7% (5.07–100.0%)	26.7% (5.07–100.0%)
Sensitivity analysis*			
Multimodal therapy (Surgery + Radiotherapy ± Chemotherapy)	11	64.8% (39.3–100.0%)	74.1% (48.4–100.0%)
Monotherapy (Surgery or radiotherapy alone , after exclusion)	4	50.0%	50.0%

*Sensitivity analysis excluded Sensitivity analysis excluded two SEER patients with unknown surgery status who received radiotherapy alone. All estimates are presented strictly for descriptive context. No formal statistical comparisons were performed.

## Discussion

Lacrimal gland adenoid cystic carcinoma is a rare but highly aggressive orbital malignancy for which high-quality epidemiological and treatment data remain limited, particularly from East Asian populations (2). This single-center retrospective case series of six consecutive patients treated at the Cancer Hospital of the Chinese Academy of Medical Sciences between 2012 and 2024 provides detailed real-world evidence on clinical presentation, modern conformal radiotherapy techniques, toxicity profiles, and survival outcomes in Chinese patients, and places these findings in the context of population-level SEER data.

Patients in the institutional cohort presented at a substantially younger median age of 31 years, with 83.3% younger than 40 years, compared with a median of 50 years in the SEER extract. This observation is consistent with Chinese multicenter clinicopathological studies of adenoid cystic carcinoma, which have shown that tumors arising in the lacrimal gland tend to occur at a younger age than those at other head-and-neck sites (11). Gender distribution was balanced. All six tumors were classified as regional stage, two-thirds were T3–T4, and involvement of the orbital wall, extraocular muscles, or optic nerve was frequent. These features align with the well-documented propensity of LGACC for early perineural and bone invasion and explain the high rate of orbital exenteration required for macroscopic clearance (2, 10, 12). Histopathology demonstrated classic adenoid cystic carcinoma features, including cytokeratin expression, CD117 positivity, a mean Ki-67 index of 25%, and MYB rearrangement in the single case tested.

Against this background of advanced local disease, every patient underwent surgical resection followed by high-dose conformal radiotherapy (66–70 Gy delivered by VMAT or TOMO). Eye-protection measures, including eyelid retractors and strict QUANTEC-based dose constraints to the lens, cornea, and optic apparatus, were systematically applied. The resulting 5-year overall survival and disease-specific survival rates of 75% compare favorably with the highest level of available evidence. In the 2024 systematic review and meta-analysis by Yan et al (12). that pooled 30 studies and 585 patients, surgery alone yielded a pooled 5-year overall survival of only 50% with a 5-year recurrence rate of 63%, whereas surgery plus adjuvant radiotherapy improved the 5-year overall survival to 67% (95% CI 61–73%) and reduced the recurrence rate to 41%. The institutional outcomes sit at the upper end of the surgery-plus-radiotherapy estimate despite a cohort composed entirely of regional-stage disease. Local control was excellent, with only one local recurrence (16.7%), and treatment-related toxicity remained predominantly grade I–II; long-term visual function was preserved in 83.3% of patients. These toxicity and functional results are consistent with modern conformal and proton series that emphasize rigorous ocular organ-at-risk sparing (3). The principal added value of the present series lies in the detailed documentation of contemporary intensity-modulated techniques (VMAT and helical tomotherapy), explicit application of QUANTEC ocular constraints, and structured reporting of long-term visual outcomes in an East Asian cohort—elements that remain sparsely reported in the existing literature.

Distant metastasis nevertheless developed in half of the patients, a rate that closely mirrors the pooled metastasis rate of approximately 35% across treatment modalities in the Yan meta-analysis (12). Conventional systemic chemotherapy was reserved exclusively for the post-recurrence setting and was never administered as part of primary therapy, reflecting both the modest objective response rates historically reported in adenoid cystic carcinoma and the absence of guideline-endorsed adjuvant regimens. Emerging multimodal strategies that incorporate neoadjuvant intra-arterial cytoreductive chemotherapy have shown promising signals in larger U.S. cohorts, with recent comparisons against SEER data reporting 5-year and 10-year disease-specific survival rates of 84% and 76%, respectively (15). Molecular characterization offers additional avenues; MYB rearrangement and activating NOTCH1 alterations, already implicated in a substantial fraction of lacrimal and salivary adenoid cystic carcinomas, represent potential therapeutic targets currently under clinical investigation (2).

Several limitations must be acknowledged. The institutional sample is small (n = 6), rendering survival estimates imprecise (wide 95% confidence intervals) and precluding formal comparative inference. The single-center, high-volume tertiary setting introduces potential selection bias. Follow-up duration was heterogeneous, with the shortest observation only 13 months. SEER treatment variables lack the granularity needed to evaluate radiation technique, dose, fractionation, ocular constraints, or toxicity, so the database serves only as a contextual benchmark. Molecular profiling was incomplete (MYB status available in only one patient). These constraints are typical of ultra-rare cancers and underscore the need for prospective multi-institutional registries that capture detailed clinical, dosimetric, and molecular data.

In summary, this series reinforces the contemporary consensus, now supported by the largest available meta-analysis, that surgery followed by carefully planned high-dose conformal radiotherapy constitutes the backbone of curative-intent therapy for LGACC. The favorable local control and ocular toxicity profile achieved with modern techniques in an Asian cohort of predominantly advanced-stage disease provide practical real-world corroboration of this approach. Persistent distant failure, however, highlights the continuing need for more effective systemic strategies, whether neoadjuvant intra-arterial chemotherapy or molecularly targeted agents directed against MYB or NOTCH1 pathways. Larger collaborative international studies that include East Asian populations and incorporate standardized molecular profiling and modern radiotherapy quality metrics are warranted.

## Conclusion

In this single-center Asian cohort of six consecutive patients with pathologically confirmed LGACC treated at CHCAMS between 2012 and 2024, patients presented at a markedly younger median age (31 years) with predominantly advanced local disease. All patients underwent surgical resection followed by high-dose conformal radiotherapy (66–70 Gy delivered by VMAT or TOMO) with systematic QUANTEC-guided ocular protection. Five-year overall and disease-specific survival both reached 75%, with excellent local control and predominantly mild toxicity; long-term visual function was preserved in 83.3% of patients. Distant metastasis occurred in 50% of cases.

These real-world outcomes support surgery plus carefully planned adjuvant high-dose conformal radiotherapy as the contemporary backbone of curative-intent therapy for advanced LGACC. Persistent distant failure underscores the need for more effective systemic strategies and for larger multi-institutional registries that capture detailed clinicopathological, dosimetric, and molecular data, particularly from East Asian populations.

## Data Availability

The original contributions presented in the study are included in the article/supplementary material. Further inquiries can be directed to the corresponding author.
